# Synthesis and Preliminary Biological Evaluation of Indol-3-yl-oxoacetamides as Potent Cannabinoid Receptor Type 2 Ligands

**DOI:** 10.3390/molecules22010077

**Published:** 2017-01-04

**Authors:** Rareş-Petru Moldovan, Winnie Deuther-Conrad, Andrew G. Horti, Peter Brust

**Affiliations:** 1 Helmholtz-Zentrum Dresden-Rossendorf e.V., Institute of Radiopharmaceutical Cancer Research, Permoserstr. 15, 04318 Leipzig, Germany; w.deuther-conrad@hzdr.de (W.D.-C.); p.brust@hzdr.de (P.B.); 2 Johns Hopkins School of Medicine, Division of Nuclear Medicine and Molecular Imaging, Department of Radiology, Baltimore, MD 21287, USA; ahorti1@jhmi.edu

**Keywords:** positron emission tomography, cannabinoid receptor type 2, binding affinity, indole

## Abstract

A small series of indol-3-yl-oxoacetamides was synthesized starting from the literature known *N*-(adamantan-1-yl)-2-(5-(furan-2-yl)-1-pentyl-1*H*-indol-3-yl)-2-oxoacetamide (**5**) by substituting the 1-pentyl-1*H*-indole subunit. Our preliminary biological evaluation showed that the fluorinated derivative 8 is a potent and selective CB_2_ ligand with *K*_i_ = 6.2 nM.

## 1. Introduction

For centuries, *Cannabis sativa* was used as medicinal plant for the treatment of pain and inflammatory processes. The discovery by Gaoni and Mechoulam [1] of Δ^9^-tetrahydrocannabinol (Δ^9^-THC)—the primary psychoactive ingredient in *Cannabis sativa*—set the stage for the identification of the endogenous cannabinoid (endocannabinoid) transmitter system in the brain. The endocannabinoid system consists of cannabinoid (CB) receptors, endogenous ligands (e.g., anandamide, 2-*O*-arachidonoylglycerol) that activate the CB receptors, and the enzymes, which are responsible for the biosynthesis (e.g., *N*-acyltransferase, diacylglycerol lipase) and deactivation (e.g., fatty acid amide hydrolases, monoacylglycerol lipases) of the endogenous ligands [2,3].

Two types of specific G_i/o_-protein-coupled CB receptors were cloned in the 1990s, termed CB_1_ and CB_2_ receptor [4,5]. CB_1_ receptors are abundantly expressed in the central nervous system, and their function has been thoroughly investigated [6]. Recently, the crystal structure of the CB_1_ receptors has been reported, providing a tool for a more accurate pharmacological investigation of this receptor subtype [7,8]. In contrast to CB_1_ receptors, the CB_2_ receptor was originally regarded as a peripheral receptor predominantly expressed in cells of the immune system [5,9,10]. However, more recent investigations proved the presence of CB_2_ receptors in glial cells (microglia and astrocytes) [11,12], and oligodendroglial [13] progenitors in vitro, as well as in microglia [14] and neuronal progenitors [12] in normal mouse brain in vivo [15].

The predominant expression of the CB_2_ receptor in cells of the immune system suggests a modulation of diverse immune functions, including cytokine production, lymphocyte proliferation, and humoral and cell-mediated immune responses [16,17]. With respect to neuroinflammation, microglia adopt a key function. Under healthy conditions, the expression of CB_2_ receptors in the brain is rather low [18]. However, inflammatory effects result in considerably increased expression levels of CB_2_ receptors [19,20]. It was found that neurodegenerative and neuroinflammatory processes in the brain related to, for example, depression, Alzheimer’s disease, multiple sclerosis, amyotrophic lateral sclerosis, or brain tumors such as glioblastoma are associated with an upregulation of CB_2_ receptor expression [18,21,22,23]. CB_2_ receptor agonists lead to a reduction of neuroinflammation and stimulation of neurogenesis. Therefore, the therapeutic potential of CB_2_ agonists is related to neurodegenerative and neuroinflammatory processes [19,24]. Moreover, the availability of CB_2_ receptors as measured with positron emission tomography (PET) [25] could potentially be used as a biomarker for neurodegenerative and neuroinflammatory processes in the brain [26,27,28,29].

Indole has been a very popular key building block for the medicinal chemistry of cannabinoid receptor ligands, and a large number of indole-derived CB_2_ selective ligands have been developed [30,31,32,33]. However, most of these ligands do not comply with the most important needs of a ligand suitable for the development of a tracer for CB_2_ brain imaging with PET; namely, high affinity towards CB_2_ (*K*_i_(CB_2_) < 1 nM) and high selectivity over CB_1_ (*K*_i_(CB_2_)/(CB_1_) > 500) [26,34]. Recently, we reported the development of a highly affine and selective ^18^F-labeled CB_2_ radiotracer (*K*_i_(CB_2_) = 0.4 nM, *K*_i_(CB_1_) = 380 nM), and proved its applicability in a mouse model of neuroinflammation [35]. However, this radioligand suffers from low metabolic stability in vivo, and therefore we redirected our focus on the structure of the literature known, highly affine *N*-(adamantan-1-yl)-2-(5-(furan-2-yl)-1-pentyl-1*H*-indol-3-yl)-2-oxoacetamide (**5**, *K*_i_(CB_2_) = 0.37 nM and *K*_i_(CB_1_) = 345 nM) [32] for the development of a CB_2_ radiotracer. In the present work, we show the synthesis and preliminary biological evaluation of fluorine-containing indol-3-yl-oxoacetamide derivatives.

## 2. Results and Discussion

**Scheme 1:**
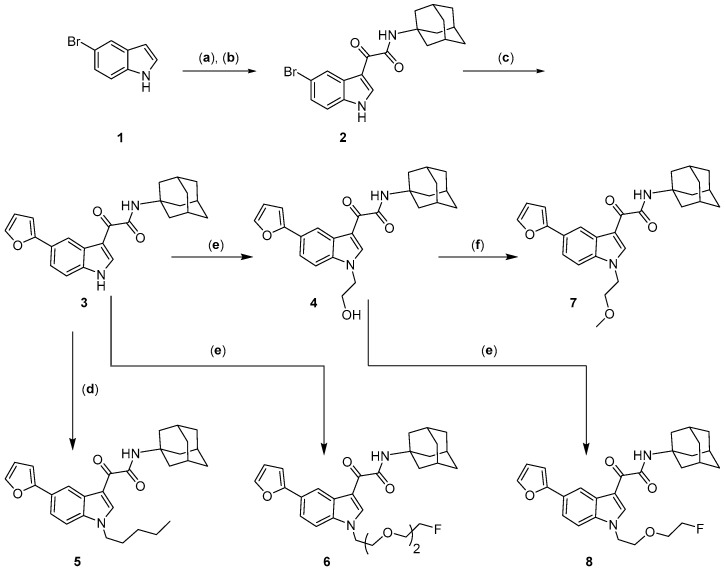
Synthesis of the key building block **3**, lead compound **5**, and ether derivatives **6**, **7**, and **8**; (**a**) oxalyl chloride, diethyl ether (Et_2_O), 0 °C to r.t., 1.5 h, 60%; (**b**) amantadine, triethylamine (Et_3_N), dichloromethane (DCM), r.t., 16 h, 82%; (**c**) 2-furanboronic acid, tetrakis(triphenylphosphine)palladium(0) (Pd(PPh_3_)_4_), Na_2_CO_3_, EtOH, reflux, 14 h, 60%; (**d**) 1-bromobutane (*n*-BuBr), tetrabutylammonium bromide (TBAB), 20% aq. NaOH, DCM, r.t., 14 h, 72%; (**e**) bromo-alkyl reagent, KOH, *N*,*N*-dimethylformamide (DMF), 90 °C, 6 h, 67%–72%; (**f**) NaH, methyl iodide (MeI) for **7** and Br(CH_2_)_2_F for **8**, DMF, 0 °C to r.t., 6 h, 85%–90% [32].

The structure–affinity relationship study which lead to the identification of compound **5** [32,36] (Scheme 1) and the work performed on the structurally related quinolinone-3-carboxamides as CB_2_ ligands [37] has proven the importance of the substitution pattern at the 5-indole position, and also the need of the lipophilic adamantane subunit. Herein, we investigate the possibility of introducing a fluorine atom by modifying the alkyl chain at the indole 1-position.

The synthesis of the key building block **3** was performed as described in the literature [32] and depicted in Scheme 1. Treatment of 5-bromoindole (**1**) with oxalyl chloride delivered the corresponding indole-3-oxoacetyl chloride in ~60% yield, which was further reacted with amantadine (adamantan-1-amine) in presence of triethylamine (Et_3_N) to give compound **2**. Next, Suzuki coupling was performed using the commercially available 2-furanboronic acid in presence of Pd(PPh_3_)_4_ and Na_2_CO_3_ to give **3**. The lead molecule **5** [32] was synthesized by treating **3** with 1-bromobutane in basic reaction condition in ~25% yield over four steps starting from **1**, as a light yellow solid. To overcome the high lipophilicity of the indole-*N*-alkyl chain [38], ether groups were introduced at this site of the molecule, and simultaneously, the influence of its length on the CB_2_ binding affinity was investigated. Thus, the glycol ether compound **6** was synthesized from **3** by using 2-(2-(2-fluoroethoxy)ethoxy)ethyl-4-methylbenzenesulfonate [39,40] as alkylating reagent in 72% yield. Similarly, alcohol **4** was synthesized by reacting compound **3** with 2-bromethanol. Alcohol **4** was further etherified via Williamson ether synthesis [41] by using methyl iodide and 2-fluoro-1-bromoethane to give compounds **7** and **8**, respectively (for ^1^H-NMR of compounds **3**, **5**, **6**, **7** and **8** see Supplementary Materials).

**Figure 1:**
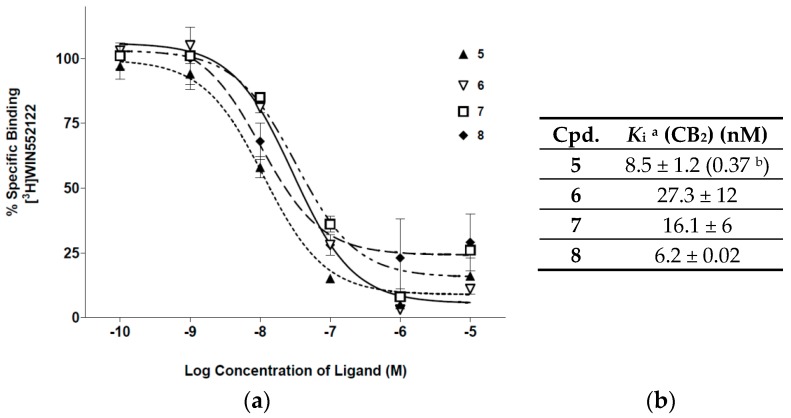
(**a**) Individual competition binding curves of compounds **5**, **6**, **7**, and **8**. Inhibition of [^3^H]WIN55.212-2 binding by increasing concentrations (100 pM to 10 µM) of compounds **5**, **6**, **7**, and **8** to membrane homogenates of CHO cells stably transfected with human CB_2_. Each value represents the mean ± standard deviation of a triplicate in a single experiment; (**b**) Binding affinity (*K*_i_) of compounds **5**, **6**, **7**, and **8** for the human cannabinoid receptor type 2 (CB_2_) receptor. ^a^ Values are means ± standard deviations of two to three experiments run in triplicate; ^b^ *K*_i_ of compound **5** as reported in [23].

The binding affinity towards CB_2_ receptors was determined in vitro by radioligand inhibition binding assays according to a recently published protocol [42,43] using cell membranes from CHO cells stably transfected with the human CB_2_ (Prof Paul L. Prather, University Arkansas for Medical Sciences, Little Rock, AR, USA), [^3^H]WIN55.212-2 as competitive radioligand (*K*_D_ = 2.1 nM) and increasing concentrations (100 pM to 10 µM) of compounds **5**, **6**, **7**, and **8** added in triplicate for each experiment. Non-specific binding was determined using 10 µM WIN55.212-2. The individual IC_50_ values were determined by non-linear curve fitting using GraphPad Prism software (version 3.0, GraphPhad, San Diego, CA, USA), and the corresponding *K*_i_ values calculated using the Cheng–Prusoff equation [44]. As shown by the *K*_i_ values in Figure 1b, we could not reproduce the reported sub-nanomolar CB_2_ affinity of the lead molecule (compound **5**) [32], but recorded a *K*_i_ value of 8.5 nM instead. In addition, these values and the individual binding curves in Figure 1a illustrate that a similar low-nanomolar affinity was observed for the fluorinated derivative **8** (*K*_i_ = 6.2 nM), while slightly lower affinities were determined for compounds **6** and **7** (*K*_i_ = 27.3 nM, and *K*_i_ = 16.1 nM, respectively). Additionally, the binding affinities towards the CB_1_ receptor were investigated for compounds **6** and **8** by using [^3^H]CP55.40 as competitive radioligand. None of the two derivatives could displace [^3^H]CP55.40 from the human CB_1_ receptors up to a concentration of 100 µM. Thus, within this series of fluoro-substituted compounds, the *N*-alkyl chain of compound **8** is most suitable to obtain high affinity binding at the CB_2_ receptor, combined with an excellent selectivity against the CB_1_ receptor subtype.

## 3. Materials and Methods

### 3.1. General Methods

All reagents were used directly as obtained commercially, unless otherwise noted. Reaction progress was monitored by thin-layer chromatography (TLC) using silica gel 60 F254 (0.040–0.063 mm) with detection by UV. All moisture-sensitive reactions were performed under an argon atmosphere using oven-dried glassware and anhydrous solvents. Column flash chromatography was carried out using E. Merck silica gel 60F (230–400 mesh) (Merck Millipore, Darmstadt, Germany). Analytical TLC was performed on aluminum sheets coated with silica gel 60 F254 (0.25 mm thickness, E. Merck, Darmstadt, Germany). ^1^H-NMR spectra were recorded with a Bruker-400 NMR spectrometer (Bruker, Billerica, MA, USA) at nominal resonance frequencies of 400 MHz, in CDCl_3_ or DMSO-*d*_6_ (referenced to internal Me_4_Si at δH 0 ppm). The chemical shifts (δ) were expressed in parts per million (ppm). High resolution mass spectra were recorded utilizing electrospray ionization (ESI) at the University of Notre Dame Mass Spectrometry facility.

### 3.2. Procedures and Compound Characterization

*N-(Adamantan-1-yl)-2-(5-(furan-2-yl)-1-(2-hydroxyethyl)-1H-indol-3-yl)-2-oxoacetamide* (**4**). 2-bromethanol (55 µL, 1.2 eq, 0.77 mmol) was added to a solution of **3** (300 mg, 1 eq, 0.64 mmol) in 5 mL *N*,*N*-dimethylformamide (DMF) at room temperature, followed by powder KOH (116 mg, 3 eq, 1.93 mmol), and the reaction mixture was warmed to 90 °C for 6 h. Saturated aqueous NaHCO_3_ solution (15 mL) and ethyl acetate (EA, 15 mL) were added, and the phases were separated. The aqueous phase was washed 2 × EA (10 mL), the combined organic solutions were dried over MgSO_4_, filtered, and concentrated by rotary evaporation. The residue was purified by column chromatography (silica, EA:Hex, 1/1) to give alcohol **4** (224 mg, 67% yield) as yellow solid. ^1^H-NMR (400 MHz, CDCl_3_): δ (ppm) *=* 1.67–1.76 (m, 6H), 2.05–2.12 (m, 6H), 2.07–2.11 (m, 6H), 2.14 (br s, 3H), 2.41 (br s, 1H), 3.96–4.06 (t, *J* = 5.18 Hz, 2H), 4.31 (t, *J* = 5.18 Hz, 2H), 6.51 (dd, *J* = 3.28, 1.77 Hz, 1H), 6.71 (dd, *J* = 3.28, 0.76 Hz, 1H), 7.35 (s, 1H), 7.40 (dd, *J* = 8.59, 0.76 Hz, 1H), 7.50 (dd, *J* = 1.77, 0.76 Hz, 1H), 7.65 (dd, *J* = 8.59, 1.52 Hz, 1H), 8.71 (dd, *J* = 1.77, 0.51 Hz, 1H), 8.99–9.10 (m, 1H). ^13^C-NMR (100 MHz, CDCl_3_): δ (ppm) = 29.36 (3C), 36.29 (3C), 41.15 (3C), 49.58, 51.84, 61.26, 104.42, 110.40, 111.72, 112.16, 118.02, 120.29, 126.77, 128.24, 135.74, 141.77, 142.03, 154.59, 161.48, 181.14.

*N-(Adamantan-1-yl)-2-(5-(furan-2-yl)-1-pentyl-1H-indol-3-yl)-2-oxoacetamide* (**5**). This compound was obtained according to literature. ^1^H-NMR (400 MHz, CDCl_3_): δ (ppm) *=* 0.86–0.94 (m, 3H), 1.28–1.44 (m, 4H), 1.69–1.81 (m, 6H), 1.93 (quin, *J* = 7.33 Hz, 2H), 2.10–2.20 (m, 9H), 4.16 (t, *J* = 7.33 Hz, 2H), 6.51 (dd, *J* = 3.28, 1.77 Hz, 1H), 6.73 (dd, *J* = 3.28, 0.76 Hz, 1H), 7.37–7.44 (m, 2 H), 7.50 (dd, *J* = 1.89, 0.63 Hz, 1H), 7.69 (dd, *J* = 8.59, 1.77 Hz, 1H), 8.73–8.78 (m, 1H), 9.04 (s, 1H). ^13^C-NMR (100 MHz, CDCl_3_): δ (ppm) *=* 13.94, 22.29, 28.99, 29.38 (3C), 29.60, 36.32 (3C), 41.21 (3C), 47.58, 51.78, 104.35, 110.43, 111.70, 111.91, 118.10, 120.13, 126.67, 128.36, 135.60, 141.62, 141.74, 154.70, 161.58, 180.97.

*N-(Adamantan-1-yl)-2-(1-(2-(2-(2-fluoroethoxy)ethoxy)ethyl)-5-(furan-2-yl)-1H-indol-3-yl)-2-oxoacetamide* (**6**). 2-(2-(2-fluoroethoxy)ethoxy)ethyl-4-methylbenzenesulfonate (40 mg, 1.5 eq, 0.15 mmol) and KOH (25 mg, 5 eq, 0.5 mmol) were added at room temperature to a solution of compound **3** (46 mg, 1 eq, 0.1 mmol) in 3 mL DMF, and the reaction mixture was warmed to 90 °C for 6 h. To the cooled down solution, 15 mL saturated aqueous NaHCO_3_ solution was added followed by 15 mL EA. The phases were separated, and the aqueous phase was separated 2 × 10 mL EA. The combined organic phases were washed with 20 mL brine, dried over MgSO_4_, and concentrated under reduced pressure. The resulting residue was purified by column chromatography (silica, EA:Hex, 1/1) to give **6** (44 mg, 72% yield) as yellow solid. ^1^H-NMR (400 MHz, CDCl_3_): δ (ppm) *=* 1.68–1.79 (m, 6H), 2.08–2.19 (m, 9H), 3.51–3.71 (m, 6H), 3.65–3.70 (m, 2H), 3.90 (t, *J* = 5.43 Hz, 2H), 4.37 (t, *J* = 5.43 Hz, 2H), 4.40–4.45 (m, 1H), 4.49–4.68 (m, 1 H), 6.51 (dd, *J* = 3.28, 1.77 Hz, 1H), 6.72 (dd, *J* = 3.28, 0.76 Hz, 1H), 7.38 (s, 1H), 7.41–7.54 (m, 2H), 7.69 (dd, *J* = 8.59, 1.77 Hz, 1H), 8.69–8.88 (m, 1H), 9.08 (s, 1H). ^13^C-NMR (100 MHz, CDCl_3_): δ (ppm) *=* 29.38 (3C), 36.32 (3C), 41.20 (3C), 47.43, 51.76, 69.66 (2C), 70.43, 70.62, 70.81, 71.02 (2C), 83.95, 104.37, 110.64, 111.70, 117.98, 120.15, 126.69, 128.21, 135.84, 141.76, 142.29, 181.21. HRMS (ESI+): *m*/*z* (%) = 523.6150 (ccd. 523.6151) [M + H]^+^.

*N-(Adamantan-1-yl)-2-(5-(furan-2-yl)-1-(2-methoxyethyl)-1H-indol-3-yl)-2-oxoacetamide* (**7**). NaH (60% suspension in mineral oil, 10 mg, 2 eq, 0.25 mmol) was added to a solution of **4** (65 mg, 1 eq, 1.12 mmol) in 2 mL DMF at 0 °C, and the mixture was stirred at 0 °C for 15 min. MeI (64 µL, 8 eq, 1.02 mmol) was added, the ice bath was removed, and the reaction was magnetically stirred at room temperature. After 5 h, the reaction was quenched by slow addition of 2 mL cold water followed by 10 mL saturated aqueous NaHCO_3_ solution and 15 mL EA. The phases were separated, and the aqueous phase was washed three times with 10 mL EA. The combined organic phases were washed with brine, dried over MgSO_4_, and concentrated under reduced pressure. The resulting material was purified by column chromatography (silica, EA:Hex, 1/1) to give **7** (55 mg, 84% yield), yellow solid. ^1^H-NMR (400 MHz, CDCl_3_): δ (ppm) *=* 1.75 (m., 6H), 2.10–2.20 (m, 9H), 3.25–3.39 (s, 3H), 3.77 (t, *J* = 5.43 Hz, 2H), 4.34 (t, *J* = 5.43 Hz, 2H), 6.46–6.55 (m, 1H), 6.68–6.78 (m, 1H), 7.39 (s, 1H), 7.40–7.45 (m, 1H), 7.47–7.54 (m, 1H), 7.69 (dd, *J* = 8.59, 1.77 Hz, 1H), 8.74 (d, *J* = 1.77 Hz, 1H), 9.01–9.10 (m, 1H). ^13^C-NMR (100 MHz, CDCl_3_): δ (ppm) *=* 30.00 (3C), 31.81, 36.43 (3C), 39.23 (3C), 47.15, 59.15, 70.73, 104.46, 110.37, 111.71, 120.26, 138.52, 141.71, 161.32, 181.22. HRMS (ESI+): *m*/*z* (%) = 447.5457 (ccd. 447.5455) [M + H]^+^.

*N-(Adamantan-1-yl)-2-(1-(2-(2-fluoroethoxy)ethyl)-5-(furan-2-yl)-1H-indol-3-yl)-2-oxoacetamide* (**8**). This compound was synthesized by employing the same procedure as for compound **7**, and it was obtained in 75% yield as yellow solid. ^1^H-NMR (400 MHz, CDCl_3_): δ (ppm) *=* 1.75 (m, 6H), 2.07–2.21 (m, 9H), 3.58–3.66 (m, 1H), 3.66–3.72 (m, 1H), 3.92 (t, *J* = 5.56 Hz, 2H), 4.38 (t, *J* = 5.43 Hz, 2H), 4.42–4.48 (m, 1H), 4.52–4.60 (m, 1H), 6.46–6.55 (m, 1H), 6.73 (dd, *J* = 3.28, 0.76 Hz, 1H), 7.37 (s, 1H), 7.46 (dd, *J* = 8.59, 0.51 Hz, 1H), 7.50 (dd, *J* = 1.77, 0.76 Hz, 1H), 7.70 (dd, *J* = 8.59, 1.77 Hz, 1H), 8.75 (dd, *J* = 1.64, 0.63 Hz, 1H), 9.07 (s, 1H). ^13^C-NMR (100 MHz, CDCl_3_): δ (ppm) = 29.38 (3C), 36.32 (3C), 41.20 (3C), 47.44, 51.79, 69.88, 70.47, 70.67, 82.31, 83.99, 104.39, 110.59, 111.70, 112.25, 118.00, 120.23, 128.21, 135.82, 141.76, 142.14, 154.65, 161.45. HRMS (ESI+): *m*/*z* (%) = 479.5627 (ccd. 479.5625) [M + H]^+^.

## 4. Conclusions

In this study, the *N*-alkyl chain of the high affinity and selective CB_2_ ligand **5** was modified for the possibility of introducing a fluorine atom. The herein developed fluorinated compound **8** retained the high affinity of the lead compound, and will be considered for the development of an ^18^F-labeled radiotracer for CB_2_ receptors imaging with PET.

## Supplementary Materials

The ^1^H NMR spectra of compounds **3**, **5**, **6**, **7** and **8** are available online at http://www.mdpi.com/1420-3049/22/1/77/s1.

## Abbreviations

CB_2_	cannabinoid receptors type 2
CB_1_	cannabinoid receptors type 1
PET	positron emission tomography
DMF	*N*,*N*-dimethylformamide
TBAB	tetrabutylammonium bromide
Et_3_N	triethylamine
CHO	Chinese Hamster Ovary
EA	ethyl acetate
Et_2_O	diethyl ether
DCM	dichloromethane
Pd(PPh_3_)_4_	tetrakis(triphenylphosphine)palladium(0)
*n-*BuBr	1-bromobutane
MeI	methyl iodide
Hex	hexane
