# Supplementary material for: Synthesis and Preliminary Biological Evaluation of Indol-3-yl-oxoacetamides as Potent Cannabinoid Receptor Type 2 Ligands
Source: Molecules. 2017 Jan 4;22(1):77. doi: 10.3390/molecules22010077 (PMC6155603; doi:10.3390/molecules22010077)
Supplement: Supplementary file 1 [file molecules-22-00077-s001.pdf]

Supplementary Materials: Synthesis and Preliminary Biological Evaluation of Indol-3-yl-oxoacetamides as Potent Cannabinoid Receptor Type 2 Ligands

Rareş-Petru Moldovan, Winnie Deuther-Conrad [1], Andrew G. Hortiand Peter Brust

^{1}H NMR of compounds **3**, **5**, **6**, **7** and **8**.

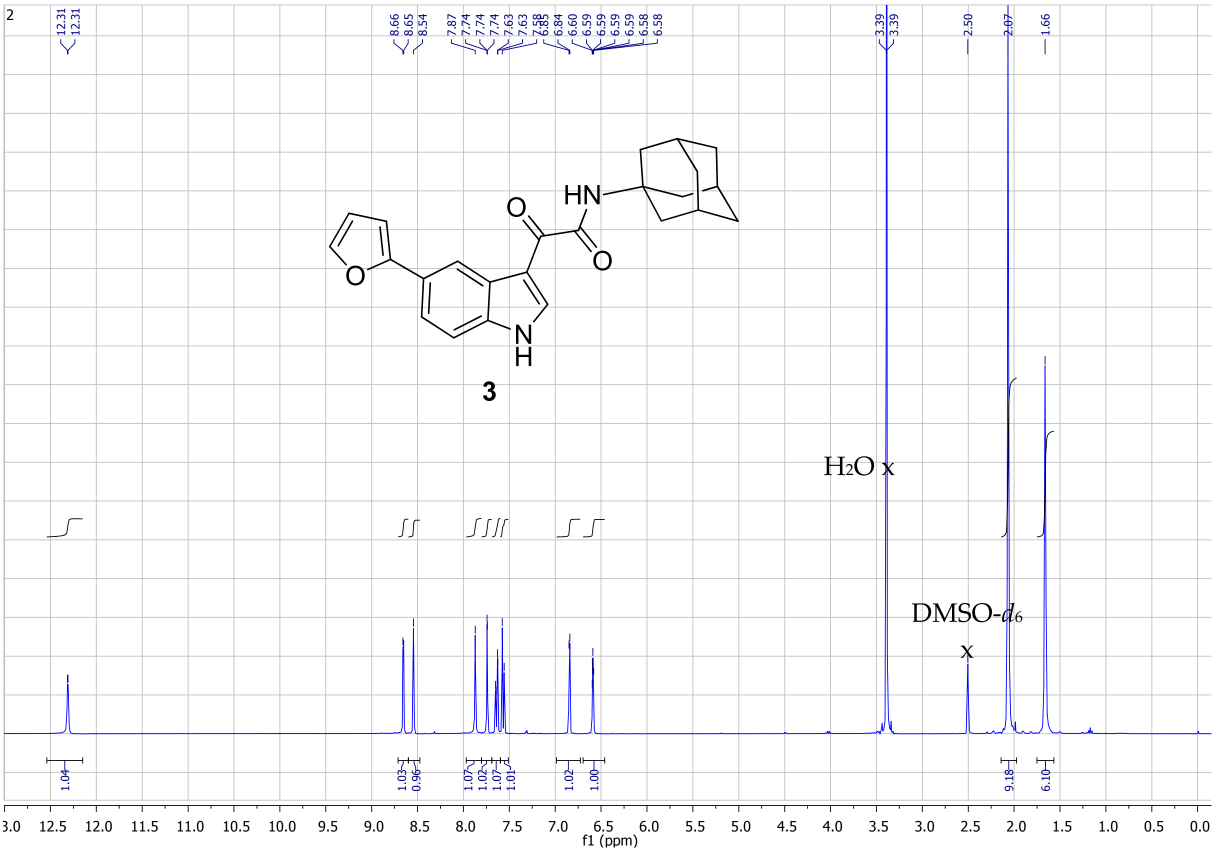

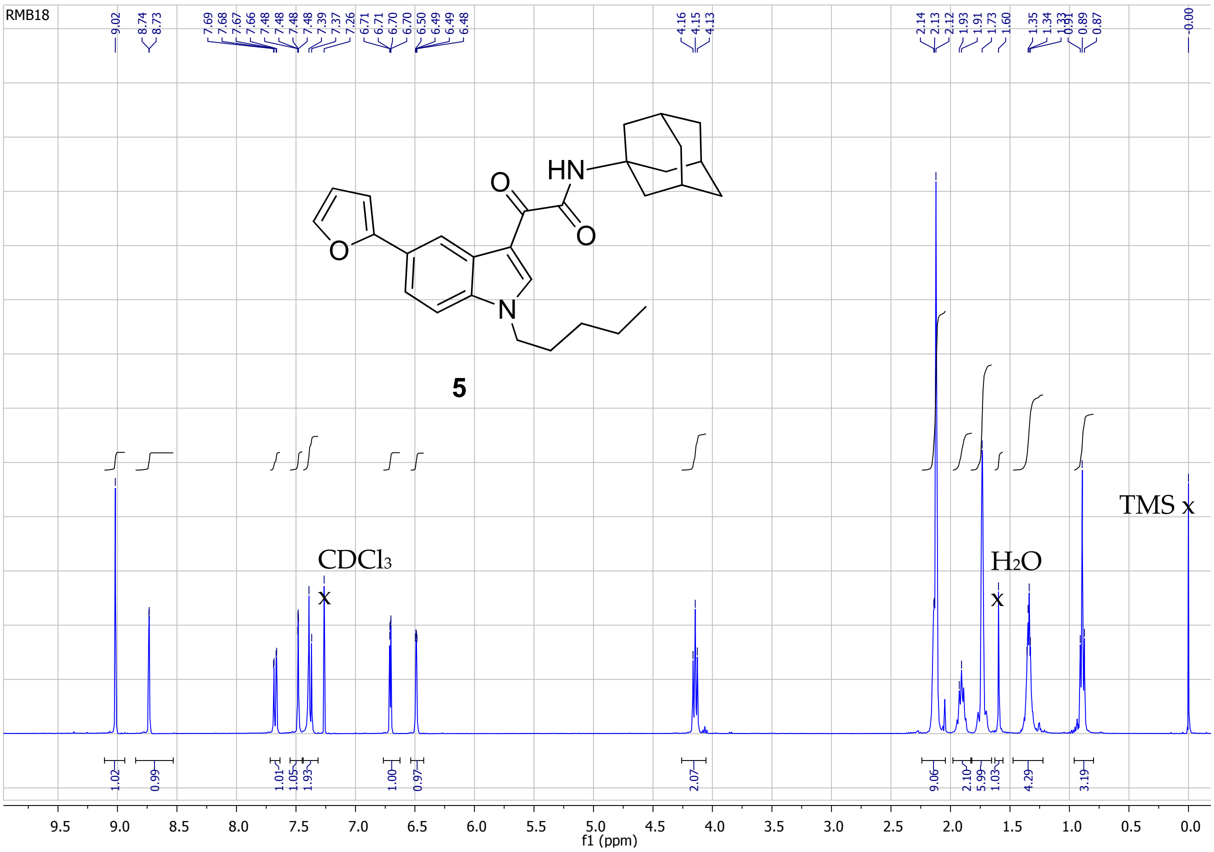
RMB18
5
CDCl3
x
H2O
x
TMS x
f1 (ppm)

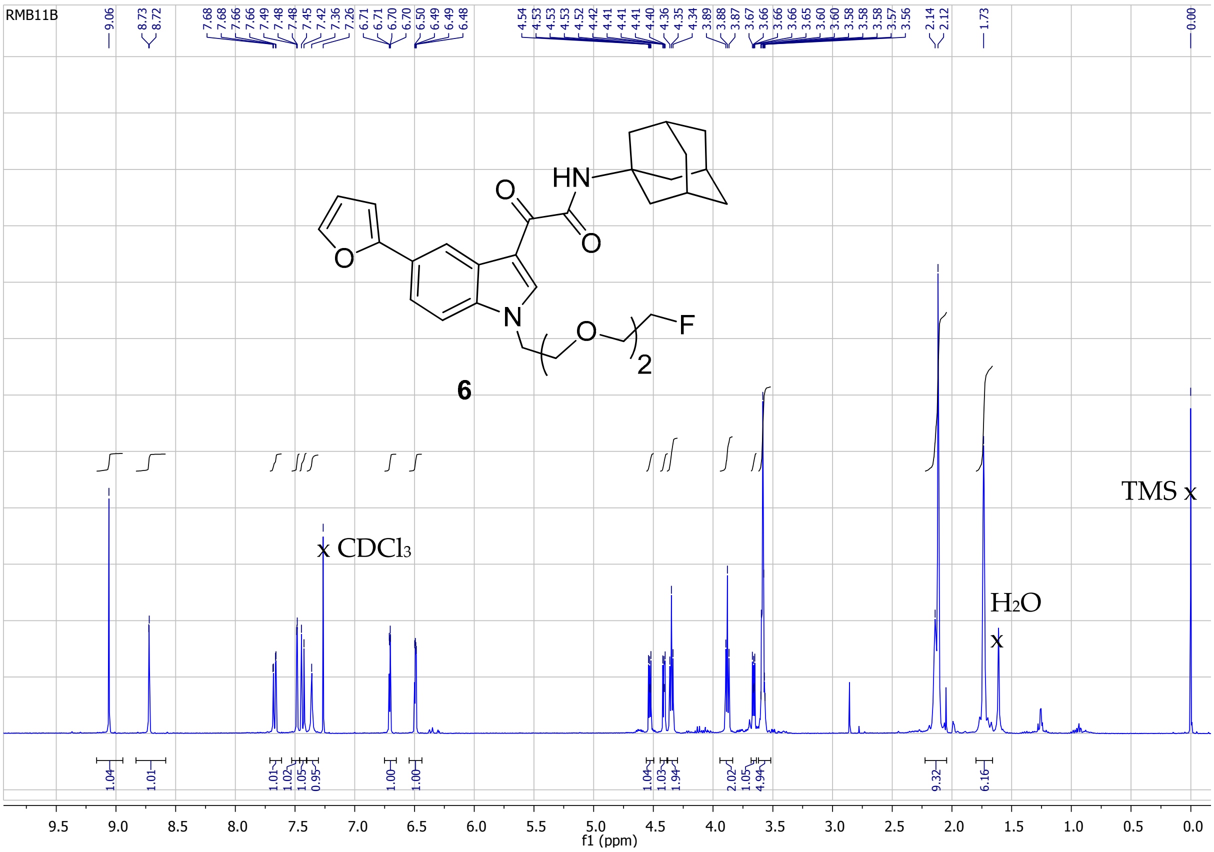
RMB11B
6
x CDCl3
H2O
x
TMS x
f1 (ppm)

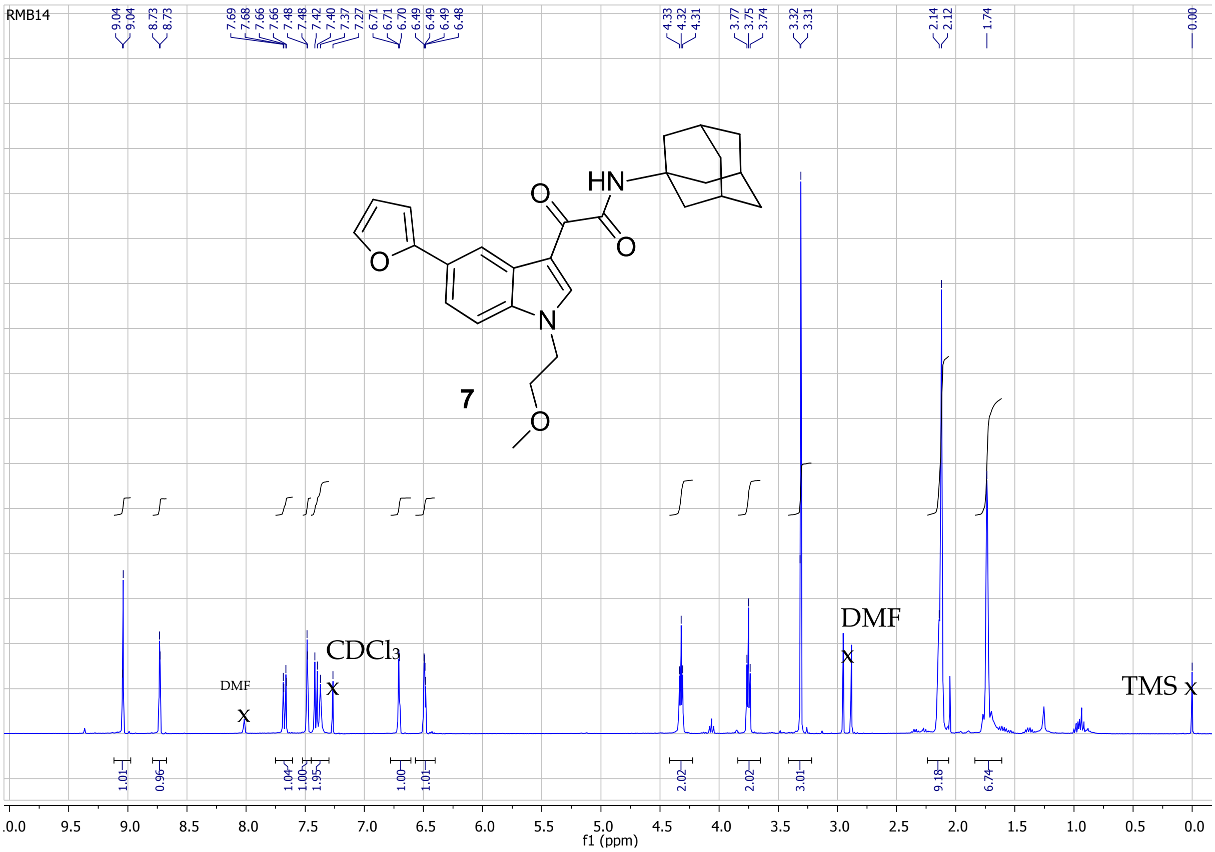
RMB14
7
CDCl3
DMF
TMS
f1 (ppm)

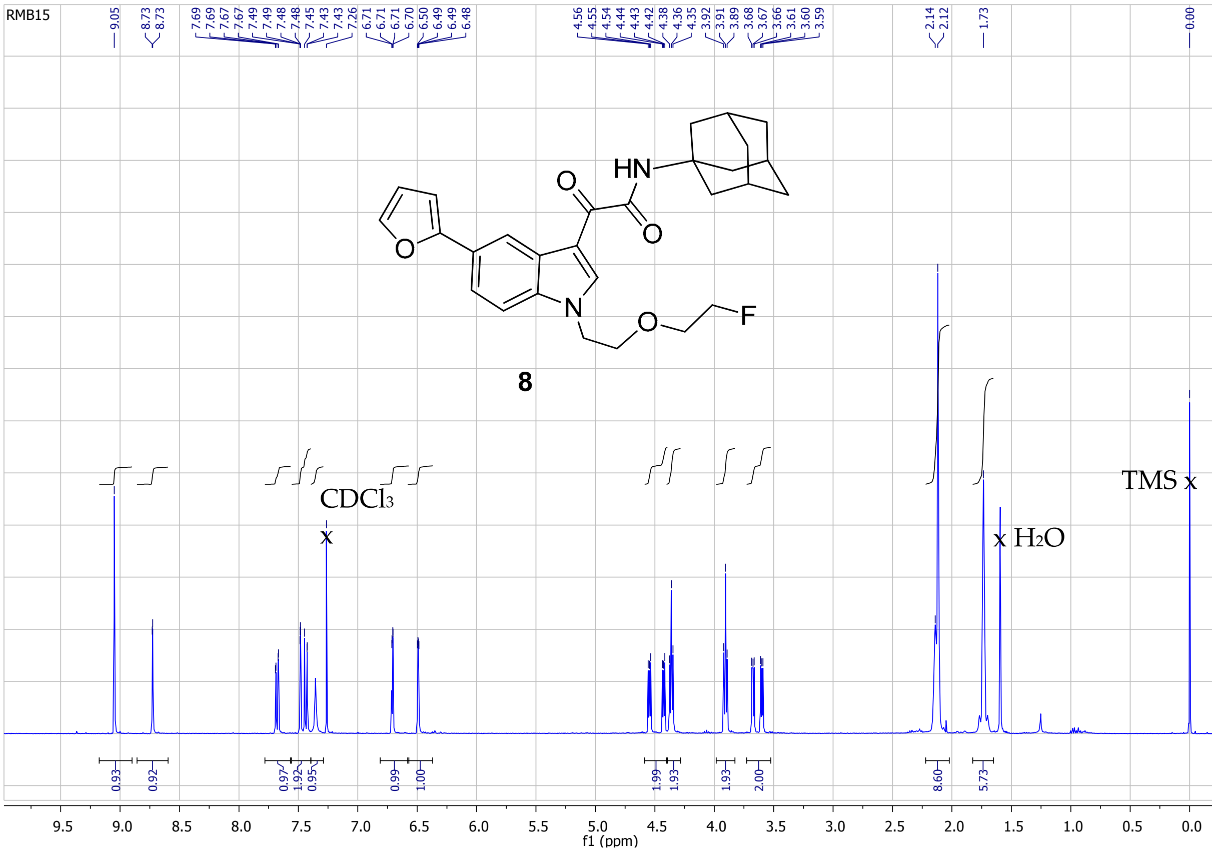
RMB15
8
CDCl3
H2O
TMS
f1 (ppm)
